# Simultaneous Distillation Extraction of Some Volatile Flavor Components from Pu-erh Tea Samples—Comparison with Steam Distillation-Liquid/Liquid Extraction and Soxhlet Extraction

**DOI:** 10.1155/2009/276713

**Published:** 2010-02-10

**Authors:** Xungang Gu, Zhengzhu Zhang, Xiaochun Wan, Jingming Ning, Chengcheng Yao, Wanfang Shao

**Affiliations:** ^1^Key Lab of Tea Biochemistry & Biotechnology, Ministry of Agriculture, Anhui Agricultural University, Hefei 230036, China; ^2^Institute of Pu-erh Tea, Yunnan Agricultural University, Kunming 650201, China

## Abstract

A simutaneous distillation extraction (SDE) combined GC method was constructed for determination of volatile flavor components in Pu-erh tea samples. Dichloromethane and ethyl decylate was employed as organic phase in SDE and internal standard in determination, respectively. Weakly polar DB-5 column was used to separate the volatile flavor components in GC, 10 of the components were quantitatively analyzed, and further confirmed by GC-MS. The recovery covered from 66.4%–109%, and repeatability expressed as RSD was in range of 1.44%–12.6%. SDE was most suitable for the extraction of the anlytes by comparing with steam distillation-liquid/liquid extraction and Soxhlet extraction. Commercially available Pu-erh tea samples, including Pu-erh raw tea and ripe tea, were analyzed by the constructed method. the high-volatile components, such as benzyl alcohol, linalool oxide, and linalool, were greatly rich in Pu-erh raw teas, while the contents of 1,2,3-Trimethoxylbenzene and 1,2,4-Trimethoxylbenzene were much high in Pu-erh ripe teas.

## 1. Introduction

Pu-erh tea is a special tea species in China and has become one of the most popular beverages in southwestern China and Southeast Asian, owing to its special flavour properties and potential healthy benefits [1]. It is originated from Yunnan province (China) through a special post-fermentative process, using crude green tea prepared from the leaves of *C. sinensis var. assamica* as original materials [2]. Because Pu-erh tea has a malty flavour and low-stimulation taste in tea infusions [1] which may fit female appetite, recent years, interest in the flavor and the healthy properties and the related scientific investigations were increasing [3, 4]. Up to now, a little information on the relationship of the chemical composition to the flavor is available. 

Volatile flavor component is one of the most important factors to influence the flavor, taste, and quality of Pu-erh tea [1], in which the contents are different from the green and black tea because of different processing procedure and variety of species and cultivar [5]. Investigation on the components in green and black tea are reported elsewhere [6], yet few on those in Pu-erh teas. In order to explore the influence of those components to flavor, taste, and quality, quantitative analysis of main volatile flavor components in Pu-erh tea is one of the key procedures. 

Sample preparation is a critical step in analytical procedure for Pu-erh tea. Compatible extraction technique can provide a convincing result for determination of target components. For analysis of volatile components or essential oils, several extraction techniques, including Soxhlet extraction [7], liquid-liquid extraction (LLE) [8], simutaneous distillation-solvent extraction (SDE) [9], solid phase microextraction (SPME) [10], and headspace microextraction (HSME) [11] and so forth, had been used to diferent matices. 

Soxhlet extraction is a classical method for decades in extraction of organic compounds from solid sample, and this apparatus has been developed to several types for special use [12]. It is considered to be a “thorough” extraction method because the organic phase cooled from condensation tube continuously passes through the target solid sample for hours. However, poor recovery commonly occurred for extraction of high-volatile or heat-labile compounds.

LLE is a conventional method for isolation of all boiling range volatile compounds, based on the compatibility of compounds with organic phase selected. The main disadvantage is solvent-consuming, tedious and, low-recovery for some target compounds [13]. 

SDE, proposed by Godefroot and so forth [14], has been widely applied to analysis of volatile components in tea samples [15]. Although low recovery has been found for extracting the most volatile or heat-labile components, this technique has achieved higher recoveries and greater repeatability of volatile or semi-volatile and heat-stable components than other isolation technique such as SPME [10] or HSME when low water temperature in the circulating system was used.

SPME and HSME are relatively new techniques that are able to address the need for concentrating the components in the headspace [10, 11]. Both of them use a small piece of fused silica, on which a liquid or solid phase, similar to a GC stationary phase, has been coated to absorb the desired components and concentrate them on the fibre. Thus the two techniques are more sensitive for the isolation of high volatile components than SDE, while less sensitive and lower repeatability for the low volatiles than SDE.

Conventionally, Pu-erh tea is condensed to a pie-like tea-biscuit in the final processing procedure. Extraction of the volatile flavor components will be different from the green and black tea. In order to select the best extraction technique for studying the volatile flavor components of Pu-erh tea, a modified SDE was evaluated for quantitative determination of the analytes using ethyl decylate as internal standard, and the two classical techniques, steam distillation-liquid/liquid extraction and Soxhlet extraction, were compared.

## 2. Experimental

### 2.1. Specimens

Pu-erh tea samples were obtained from Dayi Limited Incorporation (Menghai, Yunnan, China). The sample was dried at 40°C in electric oven for 6 h, ground to 30–60 mesh, and sealed for use.

### 2.2. Chemicals and Reagents

The reference volatile chemicals were purchased from Sigma (St. Louis, MO, USA). The stock solution was prepared by dissolving single solid/liquid standard in dichloromethane to an appropriate concentration depending on the content in Pu-erh tea. Ultra-pure water was obtained from a Milli-Q water purification system (Pall Co, IL, USA). Chromatography-grade dichloromethane was purchased from Tedia (OH, USA). The other solvents used in the test were all of analytical-grade and disdillated before use.

### 2.3. Sample Preparation

#### 2.3.1. Simultaneous Distillation-Solvent Extraction (SDE)

SDE was carried out in a microversion apparatus, as described elsewhere. Dichloromethane and ethyl decylate were employed as solvent and internal standards, respectively. For each extraction, 15 g of tea sample, 10 g sodium sulphate, 100 *μ*L internal standard solution and 300 mL ultra-pure water were placed in a 1 L flask, 50 mL dichloromethane was in a 100 mL flask, and temperature of the circulating water system was operated at 8°C. Stream distillation was stopped after 2 h, while the solvent extraction was continued for a further 15 min. The extract was concentrated to 1 mL at 10°C by a nitrogen-purge apparatus (Shanghai ANPEL Scientific Instrument Co. LTD). The concentated solution was dehydrated with anhydrous sodium sulphate for at least 12 h, of which 2 *μ*L was injected to GC or GC-MS system for analysis.

#### 2.3.2. Steam Distillation-Liquid/Liquid Extraction (SD-LLE)

For SD-LLE, 15 g of tea sample, 10 g sodium sulphate, 100 *μ*L internal standard solution and 500 mL ultra-pure water were placed in a 1 L distillation flask, respectively. The flask was connected to a condensation tube. Stream distillation was not stopped until 200 mL effluent liquid was collected. The liquid was transferred to a 500 mL of separation funnel and then extracted three times (30 mL × 3) using dichloromethane. The extracted organic phase was combined and concentrated to 1 mL at 30°C by a nitrogen-purge apparatus after internal standard was added.

#### 2.3.3. Soxhlet Extraction

15 g of tea sample containing 100 *μ*L internal standard solution were placed in Soxhlet's apparatus and 50 mL of dichloromethane in an 150 mL distillation flask. At both ends of the sample in Soxhlet's apparatus, there are 2 cm-height of Celite to help fix the sample. Extraction was carried out at 50°C for 2 hours, and extraction was concentrated to 1 mL at 30°C by a nitrogen-purge apparatus after internal standard was added. 

### 2.4. Apparatus

#### 2.4.1. GC Conditions

The concentrated extracts were chromtographed by an HP 6890 series GC system (Agilent, USA). A 30 m × 0.25 mm DB-5 quartz capillary column (Supelco, Bellefonte, PA, USA) with 0.25 *μ*m film thickness was used to resolve the volatiles. Temperature programming was as follows: initial oven temperature was set at 60°C and kept for 3 min, then raised to 200°C at a ramp of 4°C/min and kept for 2 min; to 210°C at of 1°C/min and kept for 2 min; and finally, it was raised to 270°C and kept for 7 min. Nitrogen was used as carrier gas with column head pressure at 12.26 kPa in constant pressure mode. Injection volume was 2 *μ*L. Programming split/splitless injection temperature was set at 260°C with split ratio of 10 : 1 and FID detector at 280°C.

#### 2.4.2. GC-MS Conditions

Auto system Shimaszu QP 2010 GC-MS was employed for qualitative analysis to confirm the target components. GC temperature programming. The oven temperature was set at 50°C and kept for 2 min, then raised to 60°C at a ramp of 1°C/min and kept for 2 min, to 200°C at of 4°C/min and kept for 2 min, and finally, to 270°C at 10°C/min and kept for 5 min. Carrier gas: helium. The mass spectrometry was operated at 200°C in the electron impact mode (70 eV), Scanning from m/z 40 to 600 in 0.3 s with an 0.2 s interval time of the scan; the temperature of the GC-MS interface was 280°C; the voltage of the photoelectric multiplier tube (PMT) was 200 V. The mass spectral identifications of the target components were carried out by comparing to the NIST 107 (National Institute of Standards and Technology, Gaithersburg, USA) mass spectral library as well as to Wiley 6.0 (Wiley, New York, NY, USA) mass spectral library.

## 3. Results and Discussion

### 3.1. Chromatographic Performance

**Figure 1:**
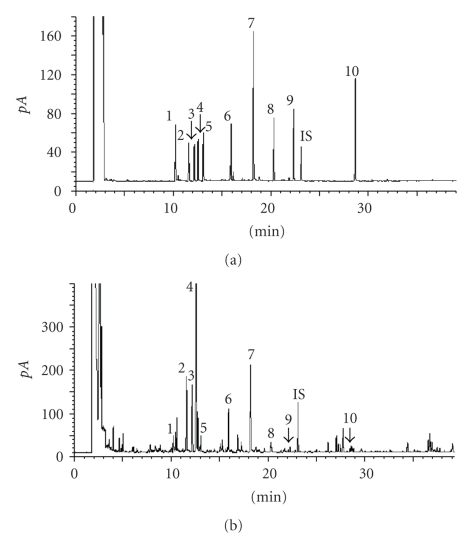
(a) Chromatogram of standard compounds, and (b) Pu-erh raw tea analyzed by GC-FID. 1 = Benzyl alcohol; 2,3 = Linalool oxide; 4 = Linalool; 5 = Phenethyl alcohol; 6 = *α*-Terpineol; 7 = Geraniol; 8 = 1,2,3-Trimethoxylbenzene; 9 = 1,2,4-Trimethoxylbenzene; IS = internal standard; 10 = Nerolidol.

**Figure 2:**
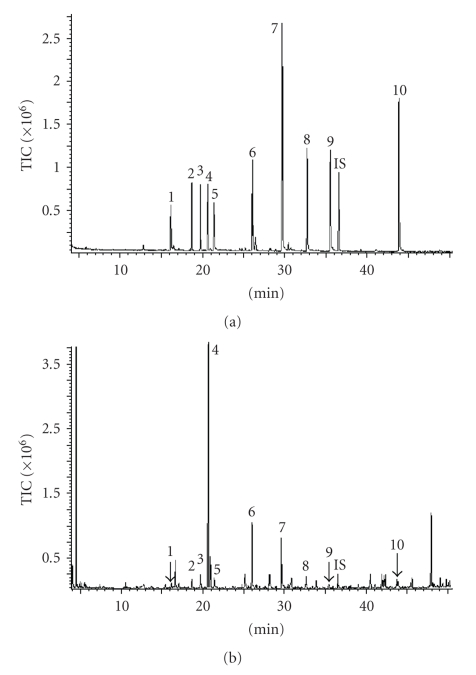
(a) TIC chromatogram of standard compounds, and (b) Pu-erh raw tea analyzed by GC-MS. 1 = Benzyl alcohol; 2,3 = Linalool oxide; 4 = Linalool; 5 = Phenethyl alcohol; 6 = *α*-Terpineol; 7 = Geraniol; 8 = 1,2,3-Trimethoxylbenzene; 9 = 1,2,4-Trimethoxylbenzene; IS = internal standard; 10 = Nerolidol.

For GC separation of volatile components in tea samples, BP-20 SGE column (polar column, 30 m × 0.25 mm i.d. film thickness 0.25 *μ*m) was commonly used for quantitative analysis [16]. In this work, DB-5 column (30 m × 0.25 mm i.d. film thickness 0.25 *μ*m) was employed to separate the target components with temperature programming described above, and the chromatograms of standard compounds by GC-FID and GC-MS was shown in Figures 1(a) and 2(a). From the chromatograms, it can be seen each peak of the target compounds was baseline separated, and separating degree between two vicinity peaks was beyond 2, confirming weak-polar capillary column can be used to separate the target components if GC separation condition was well optimized. According to GC-FID chromatogram, we calculated the relative factor of each of the standard compounds, it was shown in Table 1, and the relative factor was used to determination of the corresponding components in real Pu-erh teas.

Figures 1(b) and 2(b) show the chromatograms of Pu-erh raw tea that was obtained from GC-FID and GC-MS determination, in which the target peaks of real sample could be easily discerned and accurately quantified. The mass spectra of each target peaks in Figure 2(b) was checked by NIST or Wiley mass spectral library respectively, and campared with those in Figure 2(a), confirming that each mass spectra of the components was the same as those of the corresponding standard compound, and no interference was appeared in the target peaks.

Comparing Figures 1(b) and 2(b), we found that both GC-FID and GC-MS separation methods can be applied to quantitative analysis of volatile flavor components in Pu-erh teas. Nevertheless, quantitative determination of the target components using GC-FID was more cost-saving than doing GC-MS, so the rest determinations for all of the samples were carried out in GC-FID.

### 3.2. Extraction Solvent and Time of SDE

During SDE operation, dichloromethane and diethylether are the most suitable extraction solvents for extraction of volatile components because of their weakly polar characteristics [15, 17]. In this experimental, we select dichloromethane as extraction solvent for SDE, due to its property of low combustion, and higher boiling point (40°C) than diethylether (35°C) thus liable to storage.

Zhu et al. had applied SDE to extract volatile constitutes in green tea, confirming of 2 h was adequate to extract all the target compounds [15]. Initially, we operated SDE apparatus as Zhu et al. done, and found that there are about 6% of target components dwelling on the tea residue. This is because Pu-erh tea was condensed to a pie-like shape, and the components were more difficult to release from tea matrix although the teas had been ground to 30–60 mesh. Then we modified this method by adding 10 g of sodium sulphate to the 1 L of steam flask containing 300 mL of water, and then operating SDE device. After 2 h of SDE and a further 15 min of the solvent extraction, the tea residue was re-extracted by SDE, we found the area of the target peaks were less than 2% of the total. Obviously, boiling point of water in steam flask was enhanced by adding sodium sulphate, accelerating the releasing velocity of target compounds.

### 3.3. Recovery and Repeatability

To confirm the repeatability, parallel experimental was carried out. Five Pu-erh tea samples were extracted by SDE described above, respectively. The relative standard deviations (RSD) were in range of 1.44%–12.6%, which were shown in Table 3. To check accuracy of SDE for volatile flavor components, a known amount of standard solutions were added to aliquot of Pu-erh tea. The adding level and corresponding recoveries were listed in Table 3. The recoveries of 10 target compounds were in the range of 66.4%–109%.

### 3.4. Comparison of SDE, SD-LLE and Soxhlet Extraction

For quantitative extraction of volatile flavor components from complex matrices, common technique used is SDE. Because both liquid/liquid extraction and Soxhlet extraction are classical techniques [8, 10], in this test, the modified classical techniques were compared with SDE in extraction of the volatile flavor components from Pu-erh tea in order to select the best one for sample preparation. the results were shown in Table 2. Generally, SDE was the best one among them, because the amounts of the components extracted were greater than those by employing SD-LLC or Soxhlet for the high-volatile components such as benzyl alcohol, linalool oxide, and linalool. This may attributes to the fact that SDE was a closed and continuous extraction system, in which the target components can be “thoroughly” transferred to organic phase. Furthermore, the temperature of the circulating water in SDE was set at 8°C, reducing the lose of the high-volatile components. As for extraction of the low-volatile components in pu-erh tea, such as 1,2,4-Trimethoxylbenzene, SDE is less poor compared with Soxhlet extraction, probably due to the characteristics of high-boiling point and incompatibility with water steam. 

Although SD-LLE shares a same extraction principle with SDE, the limitation of the technique is clear in Table 2. This is due to its open steam distillation system and following step-liquid-liquid extraction. During this procedure, not only high-volatile components such as benzyl alcohol, linalool oxide, and linalool and so forth had a chance to escape from the distillation system, but the analytes hardly transferred to organic phase. However, for 1,2,3- and 1,2,4-trimethoxylbenzene, contradictory results appeared as Table 2 showed. We could not confirm whether isomerization reaction was happened between the two compounds during extraction, and further investigation was beyond the work.

Soxhlet extraction was a classical technique to extract essential oil from natural product [7]. Table 2 shows that it can almost “exhaustively” extract all the 1,2,3- and 1,2,4-trimethoxylbenzene from Pu-erh tea compared with SDE, nevertheless it is very poor for benzyl alcohol, linalool oxide, linalool, phenethyl alcohol, and geraniol. These findings indicate Soxhlet was suitable for high-boiling point (low-volatile) compounds such as essential oil [18]. As for the trace high-volatile component, SDE was appreciated.

### 3.5. Determination of Tea Samples

Using the present SDE technique following GC determination, we analyzed several tea samples, including four Pu-erh ripe tea, four Pu-erh raw tea, one green tea (Huangshan, Anhui) and one black tea (Qimen, Anhui), and the contents of the target volatile components were shown in Table 4. It can be seen that the content of high volatile components, such as benzyl alcohol, linalool oxide, linalool in raw teas are higher than those in ripe teas, these findings are mainly due to the high temperature (45–55°C) during the pile-fermentation process of ripe Pu-erh teas, leading to the lose of these components [19]. However, in raw teas, the content of 1,2,3- and 1,2,4-Trimethoxylbenzene are much lower than those in ripe teas, this is because microbes play an important role in the synthesis of the two compounds during the pile-fermentation process of ripe Pu-erh teas [20], which promote reaction procedure of methylation.

Generally, the contents of the volatile components in the green and the black teas are higher than those in Pu-erh teas, except for 1,2,3- and 1,2,4-Trimethoxylbenzene, which are not found in both of teas, probably due to lack of post-pile-fermentation process in manufacture, and linalool, lower than those in Pu-erh raw teas due to different cultivar, variety of species and processing procedure [5].

## 4. Conclusion

SDE combined GC method was constructed for determination of volatile flavor components in Pu-erh tea samples. 10 of volatile flavor components were quantitatively determined, and the recoveries and RSDs were in the range of 66.4%–109% and 1.44%–12.6%, respectively. The method was compared with SD-LLE and Soxhlet extraction, comfirming SDE was suitable for Pu-erh teas among them. Pu-erh raw tea and ripe tea samples were analyzed by the method, indicating the high-volatile components, such as benzyl alcohol, linalool oxide, and linalool, were rich in Pu-erh raw teas, while the contents of 1,2,3- and 1,2,4-Trimethoxylbenzene were much high in Pu-erh ripe teas.

## Figures and Tables

**Table 1:** Parameters of standard compounds in chromatography.

Compound	Retention time	Concentration	Peak area	Relative factor*
	(min)	(*μ*g · mL^−1^)		
Benzyl alcohol	10.23	26.88	194.1	1.0951
Linalool oxide	11.59 + 12.15	70.61	275.1	2.0296
Linalool	12.53	55.18	153.1	2.8500
Phenethy alcohol	13.07	28.24	173.1	1.2901
*α*-Terpineol	15.92	27.02	217.6	0.9884
Geraniol	18.21	99.00	626.5	1.2496
1,2,3-Trimethoxylbenzene	20.32	46.57	233.8	1.5751
1,2,4-Trimethoxylbenzene	22.35	48.22	271.8	1.4029
IS	23.10	17.30	136.8	1.0000
Nerolidol	28.65	52.56	439.7	0.9452

*Relative factor: *f *= (C*_i_* × A*_is_*)/(A*_i _*× C*_is_*)

**Table 2:** Comparison of SDE, SD-LLE and Soxhlet extraction for volatile flavor components from Pu-erh tea (*μ*g · mL^−1^).

Peak no.	Compound name	Extraction technique		
		SDE^1^	SD-LLE^2^	Soxhlet^2^
1	Benzyl alcohol	20.13	16.06	15.10
2 and 3	Linalool oxide*	61.05	33.69	23.96
4	Linalool	658.1	280.5	36.62
5	Phenethyl alcohol	18.83	17.19	10.54
6	*α*-Terpineol	59.04	25.63	51.62
7	Geraniol	70.35	30.72	4.061
8	1,2,3-Trimethoxylbenzene	13.26	4.305	11.84
9	1,2,4-Trimethoxylbenzene	3.174	4.294	5.746
10	Nerolidol	4.676	4.421	3.955

*Relative factor: *f *= (C*_i_* × A*_is_*)/(A*_i _*× C*_is_*)

**Table 3:** Recovery and repeatability.

Compound name	SDE	Spiked level	Mean	Recovery	RSD
	(*μ*g · mL^−1^)	(*μ*g · mL^−1^)	(*μ*g · mL^−1^)	(%, *n* = 3)	(%, *n* = 5)
Benzyl alcohol	20.13	13.44	33.96	103	10.3
Linalool oxide	61.05	543.3	488.1	78.6	10.7
Linalool	658.1	689.8	1321	96.1	5.73
Phenethyl alcohol	18.83	14.12	33.72	105	1.44
*α*-Terpineol	59.04	13.51	73.29	105	12.6
Geraniol	70.35	99.00	178.3	109	7.49
1,2,3-Trimethoxylbenzene	13.26	23.28	37.22	103	10.3
1,2,4-Trimethoxylbenzene	3.174	12.05	11.18	66.4	12.3
Nerolidol	4.676	5.256	8.772	77.9	8.54

**Table 4:** Contents of volatile components in tea samples (*μ*g · g^−1^).

Compound	Green tea	Black tea	Production date of Pu-erh tea							
			Pu-erh ripe tea				Pu-erh raw tea			
			2005	2006	2007	2008	2005	2006	2007	2008
Benzyl alcohol	80.7	16.8	0.132	0.229	0.329	0.263	1.15	0.634	0.587	1.34
Linalool oxide	488	13.9	3.25	4.47	5.70	3.75	4.02	4.33	5.10	4.07
Linalool	22.3	15.8	4.72	3.61	5.26	2.84	51.4	40.3	53.7	43.9
Phenethyl alcohol	54.6	14.9	0.377	0.819	0.829	0.832	1.34	0.811	0.975	1.26
*α*-Terpineol	138	2.14	1.87	1.86	2.24	1.45	0.554	1.77	0.447	3.94
Geraniol	5.28	26.3	0.412	0.300	0.597	0.442	5.26	5.10	4.72	4.69
1,2,3-Trimethoxylbenzene	ND	ND	6.35	16.4	11.3	14.1	0.707	0.645	0.815	0.884
1,2,4-Trimethoxylbenzene	ND	ND	4.15	5.62	4.08	9.91	0.211	1.34	0.099	0.212
Nerolidol	4.62	2.18	0.223	0.193	0.071	0.043	0.385	0.512	0.175	0.312
